# Awareness of Colorectal Cancer Preventive Measures Among Residents of Riyadh

**DOI:** 10.7759/cureus.70070

**Published:** 2024-09-24

**Authors:** Arezki Azzi, Khaled M Alsultan, Abdullah T Alaskar, Maan T Alhazmi, Jehad T Allhaibi, Ali T Alanazi, Sultan A Alkathami

**Affiliations:** 1 Biochemistry, Imam Mohammad Ibn Saud Islamic University (IMSIU), Riyadh, SAU; 2 General Medicine, Imam Mohammad Ibn Saud Islamic University (IMSIU), Riyadh, SAU

**Keywords:** awareness, colorectal cancer, preventive measures, risk factors for colorectal cancer, screening

## Abstract

Background

Colorectal cancer (CRC) poses a significant challenge to healthcare providers. Spreading awareness, providing preventive measures, and implementing screening programs are essential for detecting and halting the progression of the disease and decreasing the mortality associated with this type of malignancy. This study aimed to assess public knowledge and awareness of colorectal cancer preventive measures and screening programs among residents of Riyadh, Saudi Arabia, and also to design better healthcare interventions or policy development.

Methods

This cross-sectional study was carried out among residents in Riyadh, Saudi Arabia. A self-administered questionnaire was sent to participants using a Google survey. The questionnaire consisted of socio-demographic characteristics (age, gender, marital status, and occupation), a general understanding of CRC and its prevention, risk factors for developing CRC, barriers to undergoing CRC screening, and a 21-item questionnaire to assess awareness of CRC preventive measures.

Results

Of the 420 respondents, 301 (71.7%) were female, and 134 (31.9%) were between 18 and 29 years old. The overall mean awareness score was 12.5 (SD 3.05) out of 21 points. Accordingly, 240 (57.1%) were categorized as having moderate awareness, 70 (16.7%) as good, and 110 (26.2%) had poor awareness levels. Being younger, being unmarried, having heard of CRC and screening tests that detect colon cancer, and being aware of the CRC early detection campaign in Saudi Arabia were the factors associated with increased awareness.

Conclusion

There was modest awareness of CRC preventive measures among residents living in Riyadh. Significant predictors of increased awareness include younger age, being unmarried, having heard of CRC, having heard of screening tests to detect CRC, and awareness of the CRC early-detection campaigns in Saudi Arabia. Healthcare providers have a vital role in increasing awareness of CRC’s preventive measures.

## Introduction

Colorectal cancer (CRC) is one of the most frequent diseases worldwide, ranking third among the most common malignancies globally, and it is also the third and fourth leading cause of cancer-related death in men and women respectively [1]. Colorectal cancer is an adenocarcinoma that begins as a benign polyp but eventually transforms into a malignant tumor that invades and destroys healthy tissue and spreads into adjacent structures [2]. As age increases, a more severe form of the disease with a higher fatality rate emerges. Tobacco use, diabetes, and obesity are the most frequent risk factors for CRC [3]. Studies have shown that genetic factors account for around 20% of CRC cases, with first-generation relatives of CRC patients having a three-fold greater risk of CRC. Researchers suggest that a diet high in fat, animal protein, and low in cellulose may increase the risk of developing CRC [4]. Saudi Arabia has a large youthful population with CRC risk factors posing a significant health danger. Furthermore, the survival rate of Saudi CRC patients is significantly lower than that of US patients. In Saudi patients, there has been a significant increase in incidence throughout the years [5]. The most recent report by the Saudi Cancer Registry (2020) cited 1,729 cases of colorectal cancer (CRC) in the country, accounting for 12.3% of all newly diagnosed cases among Saudi nationals. According to the report, CRC afflicted 753 (55.9%) males and 594 (44.1%) females. Preventive screening initiatives are urgently needed in Saudi Arabia. Frequent screening of the population, particularly those at risk, aids in the discovery of tumors, allowing for intervention before they progress to full-blown cancer. Targeting the younger population and educating them about the proper lifestyle, screening procedures, and signs and symptoms of the disease would eventually prove beneficial in lowering the rate of cancer mortality and morbidity because CRC is known to affect young people more actively in Saudi Arabia than in Western nations [5]. The aim of this study was to evaluate the awareness of CRC prevention measures in the population of Riyadh residents and also to inform healthcare interventions or policy development.

## Materials and methods

This cross-sectional study was carried out among residents of Riyadh, Saudi Arabia, over a period of 6 months from July 2023 to December 2023, to measure the awareness of preventive measures regarding colorectal cancer (CRC).

Each participant answered a self-administered questionnaire that was sent to them using a Google survey (see Appendices). An invitation letter was distributed electronically to each participant as part of the online recruitment process. There was an integrated link to the online survey in the invitation letter. Before participants could continue filling out the electronic questionnaire, the first page of the questionnaire informed them about the study and requested their consent to participate.

The questionnaire consisted of socio-demographic characteristics (age, gender, marital status, and occupation), a general understanding of CRC and its prevention, risk factors for developing CRC, barriers to undergoing CRC screening, and a 21-item questionnaire to assess awareness of CRC preventive measures.

The sample size was found to be 385 for this cross-sectional study, based on a response rate of 50%, a confidence interval of 95%, and a margin of error of 5%. The following formula was used to calculate the sample size:

We calculated the sample size based on the following formula:

Sample Size = $(Z^2\times p\times(1-p))/c^2$

Where Z value, also known as confidence level = 1.96 for 95%

p = percentage picking a choice = 0.5 

c = confidence interval = ±5% = 0.05 

The inclusion criteria for this study were residents of Riyadh, Saudi Arabia, aged between 18 and 80 years. Exclusion criteria included participants who were below the age of 18 or above the age of 80, as well as residents of other cities. Finally, the study enrolled 420 participants, 301 (71.7%) females and 119 (28.3%) males, with 134 (31.9%) of the participants aged between 18-29 years.

The survey was divided into the following sections:

Section 1: Sociodemographic characteristics, including gender, age, marital status, and occupation. A bilingual version of the CRC preventive measures questionnaire was used to measure awareness of CRC preventive measures. It consists of 21 items; the first item assessed knowledge about CRC screening time. Items No. 2 and No. 3 were about the occurrences of CRC among males and females in Saudi Arabia. Items No. 4 to No. 8 were about the symptoms of CRC.

Section 2: The prevention of CRC recurrence and progression. Items No. 10 and No. 11 were about the effectiveness of screening in early detection and the preferred time to perform a colonoscopy. Items No. 12 to No. 18 were about the risk factors for developing CRC. Item No. 19 was about CRC pathology. Item No. 20 addressed whether family history increases the risk of having CRC or not. The last item was about the best screening tool for polyps in the colon.

The awareness of preventive measures regarding CRC was measured using a 21-item questionnaire where the correct answer for each question was coded as 1, while the incorrect answer was coded as 0. The total awareness score was calculated by adding all 21 items. A possible score ranging from 0 to 21 points was generated. The greater the score, the greater the awareness of CRC preventive measures. By using 50% and 75% as cutoff points to determine the awareness levels, respondents were considered to have poor awareness if the total score was <50%, moderate awareness if the score was 50% to 75%, and good awareness if the score was above 75%.

Statistical analysis

The data were analyzed using the software program Statistical Package for the Social Sciences (SPSS) version 26 (Armonk, New York: IBM Corporation, USA). Descriptive statistics were presented as numbers and percentages (%) for all categorical variables, while continuous variables were computed and summarized as mean and standard deviation. The differences in the awareness scores in relation to the participants' sociodemographic characteristics and general knowledge about CRC were evaluated using the Mann-Whitney U-test and Kruskal-Wallis H-test. The normality of the distribution was assessed using the Shapiro-Wilk test as well as the Kolmogorov-Smirnov test. According to the results, the awareness score followed a non-normal distribution, so non-parametric tests were applied. Values were considered significant with a p-value of less than 0.05.

## Results

In this study, we were able to collect responses from 420 enrolled participants. As seen in Table 1, 134 (31.9%) were aged between 18 and 29 years old, with females being dominant at 301 (71.7%). Participants who were married constituted 257 (61.2%). In addition, 101 (24%) were still students.

**Table 1:** Socio-demographic characteristics of participants (n=420)

Study data	N (%)
Age group	
18 – 29 years	134 (31.9%)
30 – 39 years	53 (12.6%)
40 – 49 years	104 (24.8%)
50 – 60 years	95 (22.6%)
61 – 70 years	29 (06.9%)
71 – 80 years	05 (01.2%)
Gender	
Male	119 (28.3%)
Female	301 (71.7%)
Marital status	
Single	134 (31.9%)
Married	257 (61.2%)
Divorced	17 (04.0%)
Widowed	12 (02.9%)
Occupation	
Student	101 (24.0%)
Healthcare practitioner	62 (14.8%)
Office worker	89 (21.2%)
Retired	50 (11.9%)
Unemployed	58 (13.8%)
Free Business	60 (14.3%)

In Table 2, we report that 373 (88.8%) respondents had heard of CRC, while 234 (55.7%) had heard of screening tests used to diagnose colon cancer. Among those who had heard of any screening tests, the most prominent type was colonoscopy, with 176 (75.2%) respondents aware of it. Additionally, 87 (20.7%) were aware of the CRC early detection campaign in Saudi Arabia.

**Table 2:** General knowledge about CRC and its prevention (n=420)

Statement	N (%)
Have you ever heard of CRC (colorectal cancer)?	
Yes	373 (88.8%)
No	47 (11.2%)
Have you ever heard of any screening tests that are used to detect colon cancer?	
Yes	234 (55.7%)
No	186 (44.3%)
Type of screening test you heard of ^(n=234)^	
Colonoscopy	176 (75.2%)
Fecal Testing/Stool Examination	21 (09.0%)
CT scan	15 (06.4%)
X-Ray	03 (01.3%)
Blood Carcinogenic Test/Analysis of Embryonic Cancer	03 (01.3%)
I don't know	16 (06.8%)
Are you aware of the CRC early detection campaign in Saudi Arabia?	
Yes	87 (20.7%)
No	333 (79.3%)

**Figure 1:**
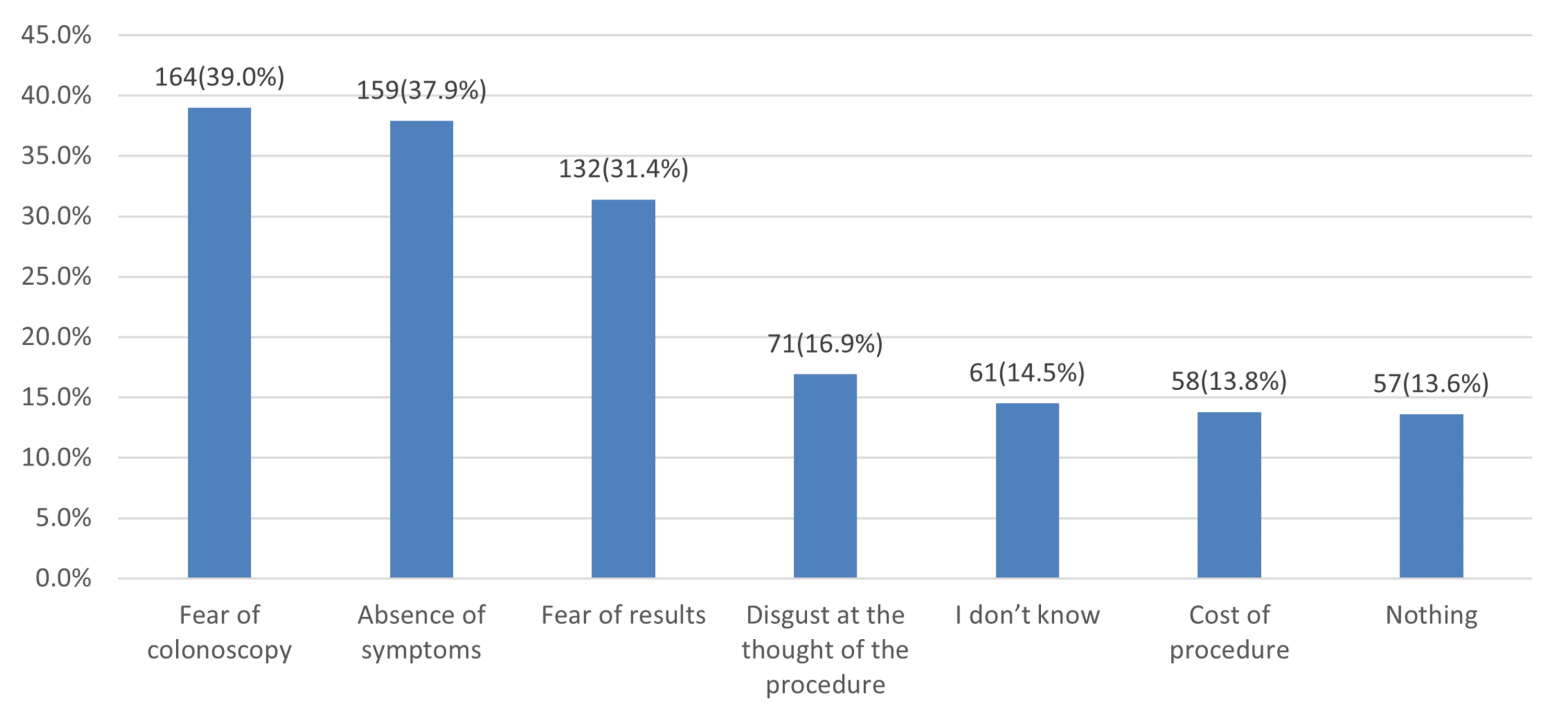
Barriers to undergoing colorectal cancer screening

In Figure 1, the most common barrier to undergoing CRC screening was fear of colonoscopy 164 (39%), followed by the absence of symptoms 159 (37.9%) and fear of results 132 (31.4%).

**Figure 2:**
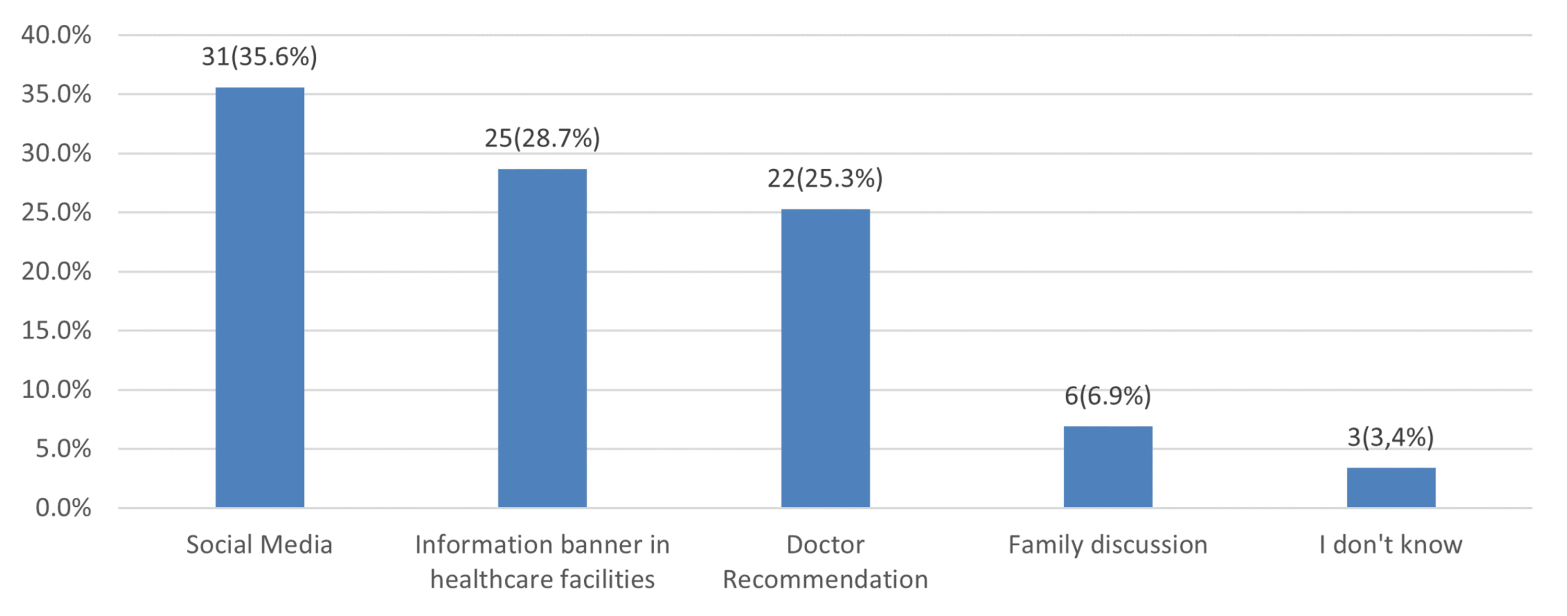
Sources of colorectal cancer early detection information

In Figure 2, among those who were aware of the CRC early detection campaign in Saudi Arabia (n=87), the most common source of CRC early detection information was social media 31 (35.6%), followed by information banners in healthcare facilities 25 (28.7%) and doctor recommendations 22 (25.3%).

**Figure 3:**
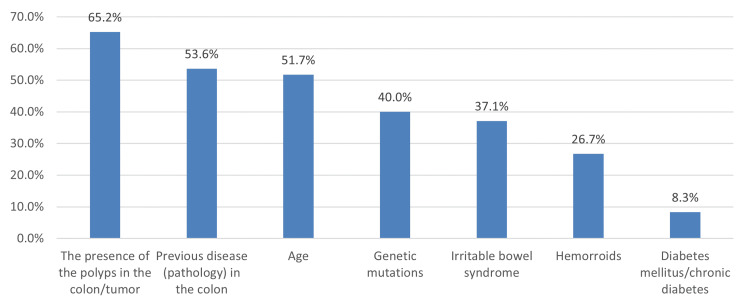
Awareness about risk factors for developing colorectal cancer

In Figure 3, the most common risk factors for developing CRC was the presence of polyps in the colon/tumor (65.2%), followed by previous disease in the colon (53.6%) and age (51.7%), while diabetes mellitus/chronic diabetes was the least (8.3%).

From the analysis of the assessment of awareness of CRC preventive measures (Table 3), it was observed that only 91 (21.7%) knew the correct age group when CRC screening should start. Only 100 (23.8%) knew CRC ranks as the most prevalent cancer among men in Saudi Arabia, and a similar proportion, 100 (23.8%), knew this for women. 277 (66%) knew that blood in the stool was the most prominent symptom of CRC, whereas 366 (87.1%) knew that vomiting was not a CRC symptom. Additionally, 347 (82.6%) believed there are ways to prevent the occurrence/progression of CRC, while 401 (95.5%) believed that screening tests increase the likelihood of detecting colon cancer early. 241 (57.4%) were aware that undergoing a colonoscopy as soon as possible is necessary once a doctor recommends it. 272 (64.8%) were aware that alcohol consumption increases the risk of developing CRC, but 405 (96.4%) believed that maintaining an average weight, and 384 (91.4%) believed that eating foods low in fat and high in fiber were not risk factors. 297 (70.7%) believed that CRC starts as a benign tumor, and 300 (71.4%) believed that a family history of CRC increases the risk of developing the disease. Additionally, 220 (52.4%) believed that the best way to detect polyps in the colon is through colonoscopy. Based on the above awareness items, the overall mean awareness score was 12.5 (SD 3.05), with poor, moderate, and good awareness levels constituting 110 (26.2%), 240 (57.1%), and 70 (16.7%), respectively.

**Table 3:** Assessment of awareness of preventive measures regarding colorectal cancer (CRC) (n=420)

Awareness statement	N (%)
When do you think CRC screening starts? [Age 41–50-year-old]	91 (21.7%)
Where does CRC rank among the most commonly occurring cancers in men in Saudi Arabia? [First]	100 (23.8%)
Where does CRC rank among the most commonly occurring cancers in women in Saudi Arabia? [Third]	100 (23.8%)
What do you think are the symptoms of CRC?	
Vomiting [no]	366 (87.1%)
Blood in stool [yes]	277 (66.0%)
Loss of appetite and weight [yes]	207 (49.3%)
Abdominal pain [yes]	195 (46.4%)
Change in bowel habits [yes]	168 (40.0%)
Do you think there are ways to prevent the occurrence or progression of CRC? [yes]	347 (82.6%)
Do you think that screening tests increase the likelihood of detecting colon cancer early? [yes]	401 (95.5%)
If your doctor recommended performing a colonoscopy, which would you prefer? [Paying and performing colonoscopy at the earliest time possible]	241 (57.4%)
Which of the following lifestyle choices increases the risk of developing CRC?	
Average weight [no]	405 (96.4%)
Eating foods low in fat and with lots of fiber [no]	384 (91.4%)
Alcohol consumption [yes]	272 (64.8%)
Physical activity and decreased exercise [yes]	237 (56.4%)
Excessive stress/anxiety [no]	236 (56.2%)
Smoking [yes]	231 (55.0%)
Eating lots of red meat [yes]	182 (43.3%)
Do you think CRC starts as a benign tumor (polyp)? [yes]	297 (70.7%)
Do you think having a family member diagnosed with CRC increases the risk of another family member developing the disease? [yes]	300 (71.4%)
What do you think is the best way to look for polyps in the colon? [colonoscopy]	220 (52.4%)
Total awareness score (mean ± SD)	12.5 ± 3.05
Level of awareness	
Poor	110 (26.2%)
Moderate	240 (57.1%)
Good	70 (16.7%)

When measuring the differences in awareness scores according to the socio-demographic characteristics of participants (Table 4), it was found that a higher awareness score was more associated with being younger (Z=2.333; p=0.020), being unmarried (Z=2.235; p=0.025), having heard of CRC (Z=5.115; p<0.001), having heard of screening tests to detect CRC (Z=8.927; p<0.001), and being aware of the CRC early detection campaign in Saudi Arabia (Z=3.757; p<0.001).

**Table 4:** Differences in the score of awareness in relation to the socio-demographic characteristics and general understanding of colorectal cancer (CRC) (n=420) ^§^P-value has been calculated using the Mann-Whitney Z-test; ^‡^P-value has been calculated using the Kruskal-Wallis H-test; ^**^Significant at p<0.05 level.

Factor	Awareness Score (21) Mean ± SD	Z/H-test	P-value ^§^
Age group			
<40 years	12.9 ± 3.12	2.333	0.020 **
≥40 years	12.1 ± 2.94		
Gender			
Male	12.7 ± 3.41	0.641	0.521
Female	12.4 ± 2.89		
Marital status			
Unmarried	13.1 ± 3.30	2.235	0.025 **
Married	12.2 ± 2.89		
Occupation			
Student	12.9 ± 3.41	3.294	0.193 ^‡^
Employed	12.5 ± 2.93		
Unemployed	12.1 ± 2.88		
Have you ever heard of CRC?			
Yes	12.8 ± 2.96	5.115	<0.001 **
No	10.3 ± 2.83		
Have you ever heard of any screening tests that are used to detect colon cancer?			
Yes	13.7 ± 2.68	8.927	<0.001 **
No	11.0 ± 2.82		
Are you aware of the CRC early detection campaign in Saudi Arabia?			
Yes	13.6 ± 2.83	3.757	<0.001 **
No	12.2 ± 3.04		

## Discussion

This study sought to determine the awareness of Riyadh residents regarding the preventive measures of CRC, a major health issue affecting both men and women. The findings of this study will be a valuable addition to the existing literature and subject to further investigation. These results provide a foundation for designing better healthcare interventions or policy development such as developing awareness campaigns throughout the country.

Level of awareness

According to our results, there was a satisfactory level of awareness among the population living in Riyadh. Nearly 60% of our subjects were deemed to have a moderate level of awareness, 70 (16.7%) had good awareness, and a little over one-fourth, 110 (26.2%), had poor awareness (total mean score: 12 out of 21 points). This is consistent with a study done in Jordan [6], where 52.9% of university students had fair knowledge of CRC symptoms, 32.8% had good knowledge, and the rest were poor (14.3%). This has been corroborated by studies done in the UAE [7] and in Trinidad [8], which reported good knowledge scores among respondents. On the contrary, several papers documented poor awareness of CRC risk factors, symptoms, and screening [9-16]. Although CRC is a widely known disease and is more common in men, the general understanding of the population regarding this type of cancer was quite low. Patients who are prone to developing CRC, particularly, adults aged 40 or older, should be more proactive in visiting their physicians if symptoms arise. Hence, a health awareness program is imperative to educate the public about the dangers of CRC.

Significant factors of awareness

Significant demographic factors associated with increased awareness include younger age and being unmarried. In Qatar [17], multiple linear regression estimates revealed that female gender, non-Qatari Arab or non-Arab nationalities, and tertiary education were identified as independent predictors of increased CRC awareness. This is comparable with studies done in Norway [18] and Poland [19], wherein women were more likely to exhibit better knowledge of CRC than men. However, in Bahrain, no significant difference was found between knowledge about CRC risk factors and symptoms in terms of age, level of education, and nationality (p>0.05). In our study, gender and occupation were also not found to have a significant relationship when compared to the score of awareness (p>0.05).

General knowledge about CRC and its influence on awareness

Incidentally, despite many of our respondents having basic information related to CRC and being aware of the screening modality used to detect CRC, 234 (55.7%), particularly colonoscopy (176 or 75.2%), only a few were aware of CRC early detection campaign in Saudi Arabia (87 or 20.7%). Nevertheless, these variables were seen to positively influence the level of awareness. While our study found a link between hearing about CRC and its screening modality in relation to awareness, Tfaily et al. found that awareness of the importance of CRC screening, past screening, and willingness to screen directly influenced awareness of risk factors and warning signs. Having a source of CRC knowledge from a family physician, undergoing regular physician check-ups, and knowing of a family member or friend diagnosed with CRC also played a role [12]. However, a study by Gede et al. noted that 27% of the adult population had not heard of CRC screening methods. They were more likely to be male, somewhat young, have a relatively low educational attainment level, and rarely see their doctor [14].

CRC symptoms awareness

Two-thirds, 277 (66%), of our respondents were confident that blood in the stool was a major symptom of CRC. Other known symptoms of CRC had lower ratings, including loss of appetite and weight (207 or 49.3%), abdominal pain (195 or 46.4%), and changes in bowel habits (168 or 40%), but most of them recognized that vomiting is not a symptom of CRC (366 or 87.1%). This is corroborated by a study done in Poland [19], where both rectal bleeding and blood in the stool were recognized as the most common symptoms associated with CRC. In Kuwait, bloody stool, lower abdominal pain, obstructed intestine, and changes in bowel habits were rated as the most common symptoms of CRC, while anemia was the least recognized (33%) [20]. Furthermore, MRI was the most frequently used diagnostic procedure (36%), followed by CT scan (31%). Interestingly, 27.7% of Kuwaitis had poor knowledge about these various diagnostic procedures. In Kazakhstan [21], half of the sample population had no idea whether CRC could occur without any symptoms and were unaware that CRC is prevalent (53.3%). However, many believed that CRC is a fatal disease but curable, with the majority (73.5%) confident that screening could provide timely and effective treatment.

Risk factors for developing CRC

Regarding their perceived knowledge about the risk factors for developing CRC, many of our subjects identified polyps in the colon/tumor (274 or 65.2%), previous disease in the colon (225 or 53.6%), and age (217 or 51.7%) as the most common factors that increased the likelihood of developing CRC. Other risk factors were less familiar among our respondents, such as genetic mutations (168 or 40%), irritable bowel syndrome (156 or 37.1%), hemorrhoids (112 or 26.7%), and diabetes (35 or 8.3%). This is almost consistent with a study done in Kuwait [20], where genetic factors and family history were also the most common risk factors for CRC, while diabetes showed minimal risk. However, in Jeddah, male gender and inflammatory bowel disease were recognized by students as potent risk factors for CRC [10].

Lifestyle risk factors

We further assessed our respondents' knowledge regarding the lifestyle risk factors of CRC. Our results showed that alcohol consumption (272 or 64.8%), followed by a lack of physical activity (237 or 56.4%), smoking (231 or 55%), and eating lots of red meat (182 or 43.3%) were labeled as the major lifestyle risk factors associated with CRC. Additionally, most of our population believed that maintaining a normal weight (405 or 96.4%) and eating foods low in fat and high in fiber (384 or 91.4%) were not risk factors. These results are almost consistent with a study on teachers' knowledge [13], which suggested that drinking more than one glass of alcohol per day and regularly consuming red meat could increase the risk of developing CRC. However, 40% of teachers believed that eating less than five portions of fruits and vegetables could also lead to developing CRC. This corroborates Rocke's study, which suggested that frequent/excessive alcohol consumption and low physical activity are risk factors for CRC, while prudent dietary patterns were identified as protective factors [8].

Sources of CRC early detection information

Sources of CRC information are crucial when gauging the population's understanding of the disease. In our study, among those who were aware of the early detection campaigns in Saudi Arabia (n=87), the most frequent source of information was social media (31 or 35.6%), followed by information banners (25 or 28.7%) and doctor's advice (22 or 25.3%). Few participants mentioned family discussions as their source of information (6 or 6.9%). In the UAE, respondents also indicated media as the most common source of CRC information [22], while in Norway, the National Public Workforce and the Norwegian Cancer Society were shown to be the most trustworthy entities for gaining valuable information related to CRC [18].

Barriers to undergoing CRC screening

The barriers to undergoing CRC screening are important to discuss. Our population identified fear of colonoscopy (164 or 39%), absence of symptoms (159 or 37.9%), and fear of results (132 or 31.4%) as the most prominent barriers to undergoing CRC screening. Disgust at the thought of the procedure (71 or 16.9%) and the cost of the procedure (58 or 13.8%) were secondary barriers. This mirrored the results of Al-Hejeili et al., who reported that fear of the procedure, fear of the results, and the absence of clinical symptoms were the most important barriers to seeking screening [23]. However, in a study conducted by Tfaily et al., the evaluation of participants' understanding of screening techniques and disease misconceptions were detected as the most significant barriers to CRC screening, which did not coincide with our findings [12].

Limitations

There are some limitations that should be taken into consideration when interpreting the results. Firstly, the study design, which is cross-sectional, could be prone to some forms of recall biases and inaccuracies. Secondly, this study was conducted in one city, which makes generalization to the Saudi population difficult. Thirdly, the sample may be biased towards individuals who are more active on social media and have a higher level of education. 

## Conclusions

There was a moderate level of awareness of CRC prevention measures among Riyadh's residents. Younger participants who were not married and had previously heard of CRC and its screening methods tended to be more aware of CRC preventive measures than the rest of the population. There is a need to increase awareness of CRC among the population living in Riyadh, Saudi Arabia. One of the most important aspects of raising awareness is eliminating the identified barriers to CRC screening. Hence, healthcare sectors should take collective measures to fill the awareness gap and alleviate the barriers and misconceptions surrounding it.

## Appendices

Questionnaire

Demographics 

1) Place of residence?

Riyadh

Other

2) How old are you?

18-29

30-39

40-49

50-60

61-70

71-80

80<

3) what is your gender?

Male

Female

4) Marital status?

Married

Single

Widow

Divorced

5) What is your occupation?

Student

Health practitioner

Office worker

Retired

Unemployed

Other

6) Have you ever heard of CRC (colorectal cancer)?

Yes

No

7) Have you ever heard of any screening tests that are used to detect colon cancer?

Yes

No

8) If you Answered yes to the previous question, what type of screening test have you heard of?

Colonoscopy

Fecal testing

CT scan

X-ray

Blood carcinogenic test

Don’t know

9) When do you think CRC screening starts?

20-30

31-40

41-50

51-60

61-70

10) Which of the following could prevent you from performing a screening? (more than one answer is acceptable). 

Fear of colonoscopy

Fear of results

Disgust at the thought of the procedure

Cost of the procedure

Absence of symptoms

Nothing

Don’t know

11) Are you aware about CRC early detection campaign in Saudi Arabia?

Yes

No

12) if you answered yes, through which way did you get the information?

Doctor recommendation

Information banner in healthcare facilities

Social media

Family discussion

I don’t know

13) Where does CRC rank among the most commonly occurring cancers in men in Saudi Arabia?

First

Third

Seventh

Tenth

Don’t know

14) Where does CRC rank among the most commonly occurring cancers in women in Saudi Arabia?

First

Third

Seventh

Tenth

Don’t know

15) What do you think are the symptoms of CRC?  (more than one answer is acceptable)

Blood with stool

Vomiting

Loss of appetite and weight

Change in bowel habits

Abdominal pain

I don’t know

16) Do you think there are ways to prevent the occurrence or progression of CRC?

Yes

No

17) Do you think that screening tests increase the likelihood of detecting colon cancer early?

Yes

No

18) If your doctor recommended performing a colonoscopy, which would you prefer?

Paying and performing colonoscopy at the earliest time possible

Waiting until your free appointment

I prefer not to answer

19) Which of the following lifestyle choices increases the risk of developing CRC?  (more than one answer is acceptable).

Smoking

Alcohol consumption

Eating lots of red meat

Physical inactivity and decreased exercise

Eating foods low in fat and with lots of fiber

Excessive stress

Average weight

20) Which of the following increases the risk of CRC?  (more than one answer is accepted)

Age

The presence of polyps in the colon

Genetic mutations

Diabetes mellitus

Irritable bowel syndrome

Previous disease (pathology) in the colon

Hemorrhoids

21) Do you think CRC starts as a benign tumor (polyp)

Yes

No

22) Do you think having a family member diagnosed with CRC increases the risk of another family member developing the disease?

Yes

No

23) What do you think is the best way to look for polyps in the colon?

Colonoscopy

CT scan

X-ray

Clinical examination

Don’t know
